# Tips and Tricks—3 Use Cases of Interdisciplinary Knowledge Transfer in Minimally Invasive Pediatric Surgery

**DOI:** 10.3390/children9091270

**Published:** 2022-08-23

**Authors:** Mareike Grosshauser, Tim Ohletz, Valérie Oesch, Cécile Olivia Muller

**Affiliations:** 1 Department of Paediatric Surgery, Cantonal Hospital Aarau, Tellstrasse 25, 5000 Aarau, Switzerland; 2 Department of Radiology and 3D Lab, Cantonal Hospital Aarau, Tellstrasse 25, 5000 Aarau, Switzerland

**Keywords:** cholecystectomy, cholangiography, AeroStat, magnet, magnetic retriever, magnetic rod, MRI 3D model, anorectal malformation

## Abstract

In the pediatric surgical environment, we can enrich our knowledge and improve our skills through interdisciplinary knowledge transfer in exchange with other surgical or even non-surgical disciplines. To demonstrate this, we present in this article three patient cases of method transfer enabling new techniques in minimally invasive pediatric surgery. 1. The somewhat modified application of the AeroStat rigid tip laparoscopic cholangiography catheter during the single-step laparoscopic cholecystectomy facilitates the safe intraoperative cholangiography with common bile duct flushing. 2. A magnetic rod is used during laparoscopic enterotomy to locate and retrieve ingested magnets. 3. Using a patient-specific MRI 3D model representing a syndromic high anorectal malformation improves surgical planning and parental education.

## 1. Introduction

Successful treatment of pediatric patients requires close cooperation between physicians and the patient, but also the parents, with a special focus on safety. However, as in any other field, pediatric surgeons must always integrate the latest evidence-based methods and therapies into pediatric surgical techniques in order to provide the best possible care. Nowadays, this includes efficient and minimal-invasive methods such as laparoscopy and the latest imaging techniques. These are used to plan complex surgeries and communicate with the patients and the parents. Aside from the medical literature, we can also benefit from transferring best practices and knowledge from other surgical specialties to stay up to date.

In this article, we will present three use cases, including techniques we picked up from our colleagues in visceral surgery and neurosurgery, and also show how we applied the latest developments in imaging for pediatric surgical planning.

In the first case, we explain the slightly modified application of a tool from visceral surgery: the AeroStat rigid tip laparoscopic cholangiography catheter with a percutaneous introducer for an easy and safe intraoperative cholangiography with common bile duct flushing during cholecystectomy.

In the second case, we describe the use of a tool from neurosurgery: using a magnetic rod designed to find and retrieve metal clips during brain surgery and metal splinters during spine surgery that facilitates the intra-abdominal laparoscopic search of multiple ingested magnets.

In the third case, we describe the use of a patient-specific MRI 3D model representing a syndromic high anorectal malformation with sacral meningocele to improve the planning of the complex surgery and parent education.

## 2. Case Presentations

### 2.1. Case 1

A 15-year-old boy with Gilbert syndrome (homozygous duplication of the promotor of UGT1A1-gene, not known at first presentation) referred with unclear jaundice with aggravating episodes of abdominal pain and nausea for half a year was diagnosed with a pronounced cholecystolithiasis with a large gallbladder almost completely filled with concretions and two smaller concrements (3 mm) in the neck of the gallbladder without cholecystitis or enlargement of the bile ducts and with an unremarkable pancreas.

Laboratory chemistry studies revealed cholestasis with a total bilirubin of 254 μmol/L and a direct bilirubin of 17.6 μmol/L with an ALAT 165 U/L, ASAT 61 U/L. In the synopsis of the boy’s history and sonographic evidence of cholecystolithiasis, his symptoms were interpreted as past colics during stone passage, and the indication for elective cholecystectomy was made to prevent further painful episodes or the occurrence of complications of cholecystolithiasis.

**Figure 1:**
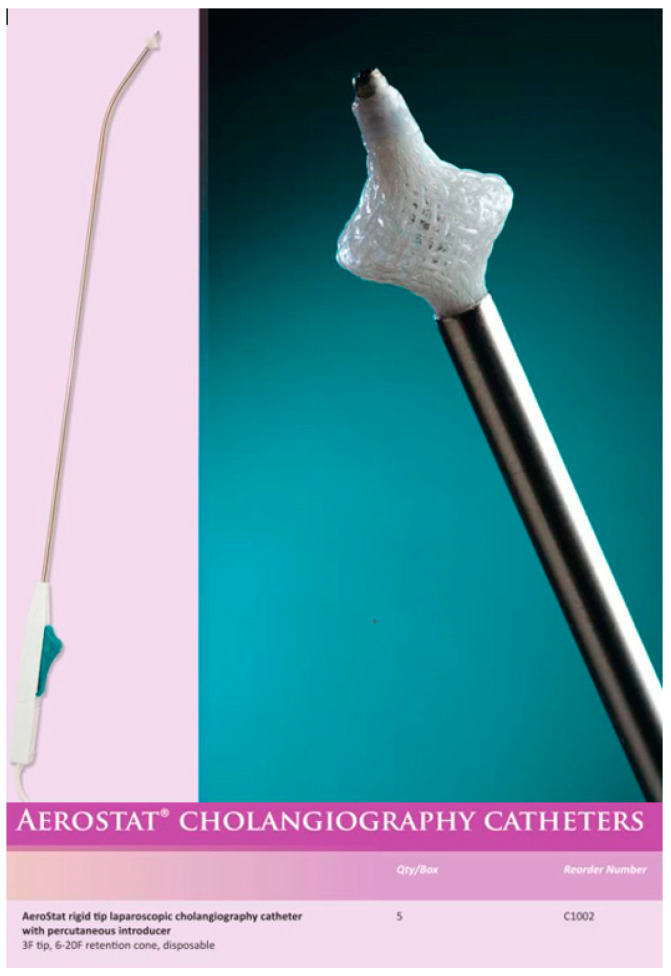
AeroStat rigid tip laparoscopic cholangiography catheter.

**Figure 2:**
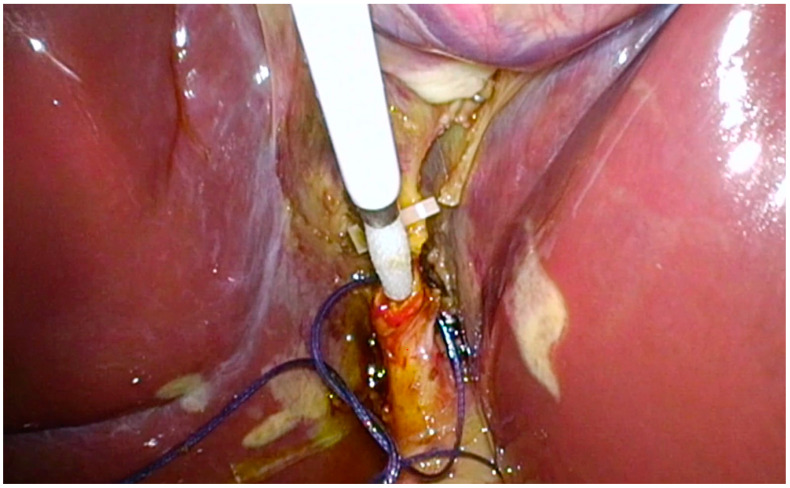
Insertion of the AeroStat cholangiography catheter and prepared ligature (see also Video S1).

During laparoscopic cholecystectomy in routine technique (Video S1) with the patient placed in the French position, operator between the legs, with a transumbilical minilaparotomy for a 10 mm trocar and three 5 mm trocars in the right lower quadrant, left upper quadrant, and infrasternal and after dissection of Callot’s triangle, identification of the cystic duct and cystic artery and clipping and cutting of the artery, the AeroStat rigid tip laparoscopic cholangiography catheter was prepared (Figure 1). The percutaneous introducer was set under direct vision through the abdominal wall near the cystic duct. In addition, to prevent the catheter’s future dislocation, a ligature Vicryl 4-0 was prepared for the stump of the cystic duct leading to the common bile duct. A clip was placed on the gallbladder side of the cystic duct, which was then incised but not completely transected. Then, the rigid shaft of the AeroStat cholangiography catheter with its 3F rigid tip was inserted into the cystic duct (Figure 2). The prepared ligature was tightened around the cystic duct with the catheter inside. The 6–20F expandable retention cone for the self-anchoring mechanism was set by a one-handed activation of the catheter’s ratcheting handle.

**Figure 3:**
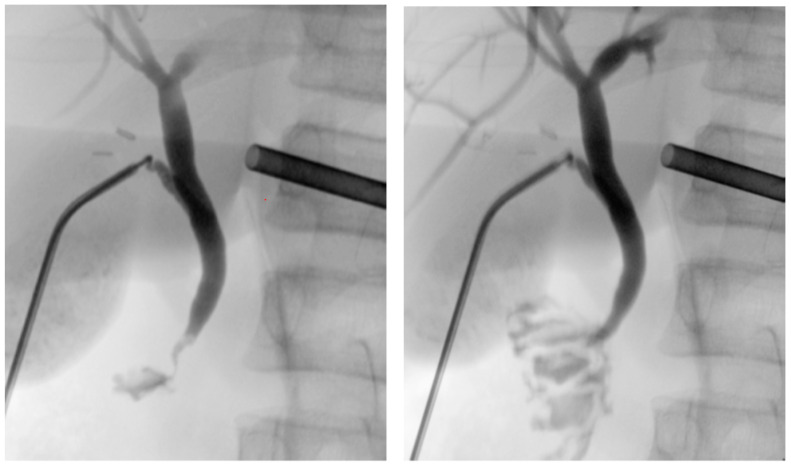
Cholangiography before and after flushing out a small stone in the common bile duct.

The patient was placed in a reverse Trendelenburg position, and the cholangiography was performed using fluoroscopy for dynamic visualization of the biliary tree. This showed the intrahepatic bile ducts as well as the common bile duct without dilatation and a free passage of the contrast medium into the duodenum. During irrigation of the common bile duct with normal saline serum, a small stone in the common bile duct could easily be flushed out (Figure 3). After extraction of the cholangiography kit, the cystic duct was closed by placing two clips on the permanent side and then divided. There was no intraoperative bile leak.

The postoperative course was uncomplicated. The patient was discharged home on the second postoperative day with compensated pain in good general condition. The progress at home was unremarkable, and in the clinical check-up one month after surgery, the boy was fine, without complaints and with normal appetite and bowel movements. He and his parents were informed that the diagnosis of Gilbert syndrome had been confirmed in the genetic tests, and genetic counseling was provided.

### 2.2. Case 2

**Figure 4:**
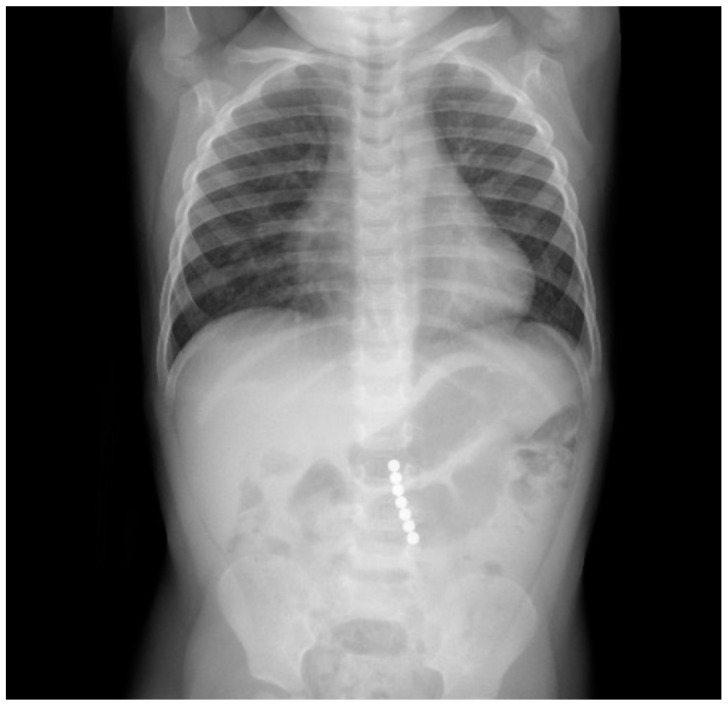
Conventional radiography showing the ingested magnets.

A 2-year-old boy was taken into the emergency department 3 hours after ingestion of 7 magnets of 5 mm diameter, which belonged to a building toy. During ingestion, the boy showed no signs of dyspnoea, coughing, or gaging, and afterward, no problems in swallowing. During the physical examination, the abdomen was soft without tenderness on palpation or signs of organomegaly with regular bowel sounds and unremarkable cardiopulmonary examination. An X-ray revealed 7 contiguous, round, radiopaque foreign bodies in projection to the mid-abdomen at LWK 3–5 level with a total length of 3.8 cm and with an adjacent, low-prominence, gas-filled loop of bowel with a maximum diameter of 3 cm with otherwise nonspecific bowel gas distribution. There was no free gas intra-abdominally. The conventional radiological image (Figure 4) was interpreted as a projection of the magnets onto the small intestine, whereby one distance between the beads appeared larger than the others and gave the impression of pinched tissue, giving the indication for diagnostic laparoscopy and foreign body extraction to avoid the occurrence of small intestine or colon perforation.

**Figure 5:**
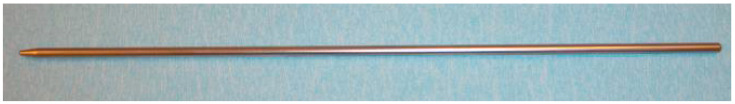
Magnetic retriever.

During laparoscopy in routine technique (Video S2) with the patient in the French position and with a transumbilical minilaparotomy for a 10 mm trocar and two 5 mm trocars in the right and left lower quadrant, the all-round view showed normal upper and lower abdominal organs. Through the 10 mm trocar, a magnetic rod (Figure 5) was inserted intra-abdominally, and the entire small intestine was scanned starting from Bauhin’s valve.

**Figure 6:**
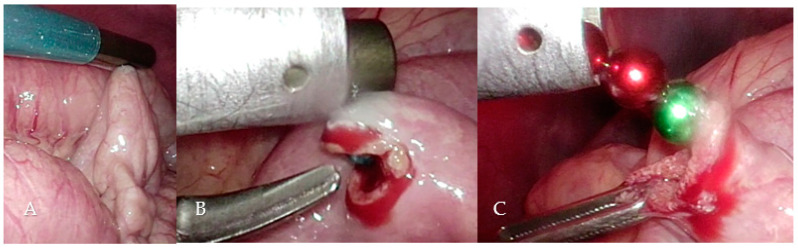
Magnetically adherent small bowel loop (**A**), enterotomy with visible blue magnetic ball (**B**), removal of the magnetic balls (**C**) (see also Video S2).

The magnets could be localized in the mid-small bowel region during this procedure just after a few minutes (Figure 6A). A laparoscopic enterotomy was performed, and the 7 magnets were attracted and fixed by the magnetic rod and extracted directly through the trocar (Figure 6B,C). The enterotomy was closed with three Vicryl 4-0 sutures.

The postoperative course was without complications with the continuation of antibiotic prophylaxis with cefuroxime and metronidazole for 72 h and a steady dietary build-up from the second postoperative day with discharge home on the fifth postoperative day. The postoperative course was uneventful.

### 2.3. Case 3

**Figure 7:**
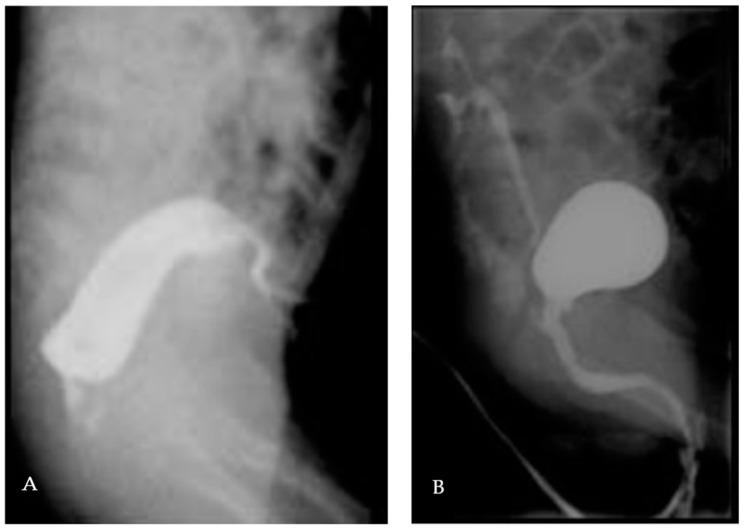
(**A**): Colon contrast imaging with recto-prostatic fistula. (**B**): MCUG with recto-prostatic fistula and vesicoureteral reflux grade III right.

A premature boy (33 weeks of gestation) was diagnosed at birth with a syndromic anorectal malformation. After performing a separate, double-barrelled left colostomy, the further diagnostic workup showed a recto-prostatic fistula (high anorectal malformation, Figure 7A), sacral agenesis, a sacral meningocele, left renal agenesis associated with cysticseminal vesicles, a vesicoureteral reflux grade III right (Figure 7B), an inguinal testis left, and microcephaly. The genetic results are still pending.

**Figure 8:**
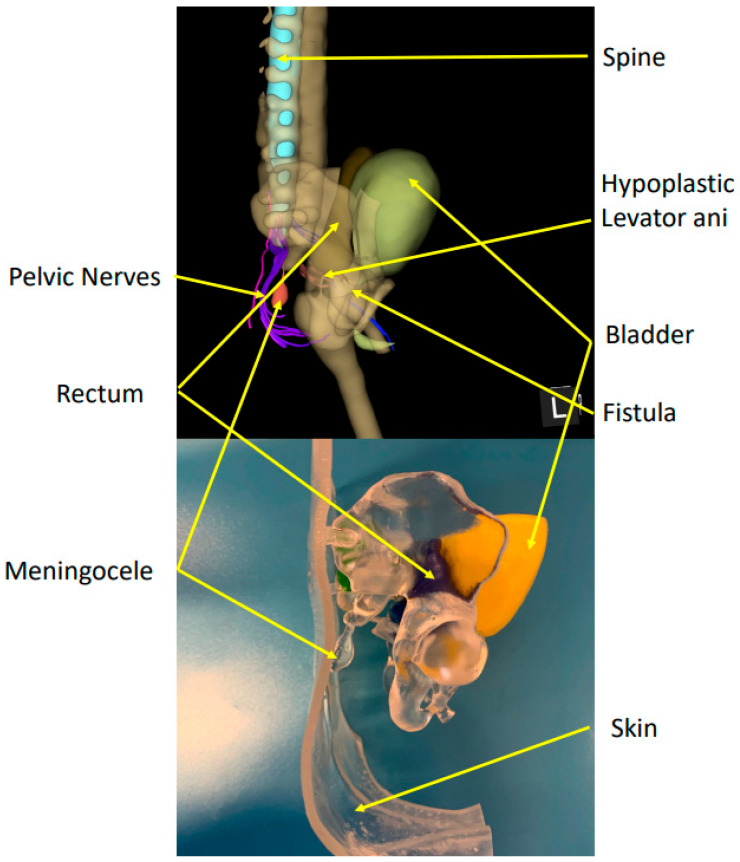
3D Segmentation in 3D Slicer (**top**) and 3D printed model (**bottom**).

In this case, we present the use of a 3D MRI reconstruction of the pelvis and spine, which was made at the age of 5.5 months and at a weight of 4.9 kg, to help with surgical planning and communication with the parents (see Figure 8). The segmentation was performed manually using the software Slicer3D by our radiologist, who then printed and colored the 3D model of the skeleton and pelvic organs in the 3D Laboratory of our hospital. The skin was also printed in flexible plastic in order to resemble reality (Figure 6). This printed 3D model allowed us to highlight the proximity of the meningocele and the recto-urethral fistula and imagine that a combined operation may be possible using two separate skin incisions: one for the meningocele excision and one for the laparoscopic assisted anorectal pull-through. This 3D model also helped us to communicate with the parents, allowing an easier understanding of the surgical possibilities and risks in this complex anatomical case. We included this patient in our ongoing study about 3D and communication, and the used questionnaires showed a clear improvement in the parent’s comprehension after the 3D model presentation.

**Figure 9:**
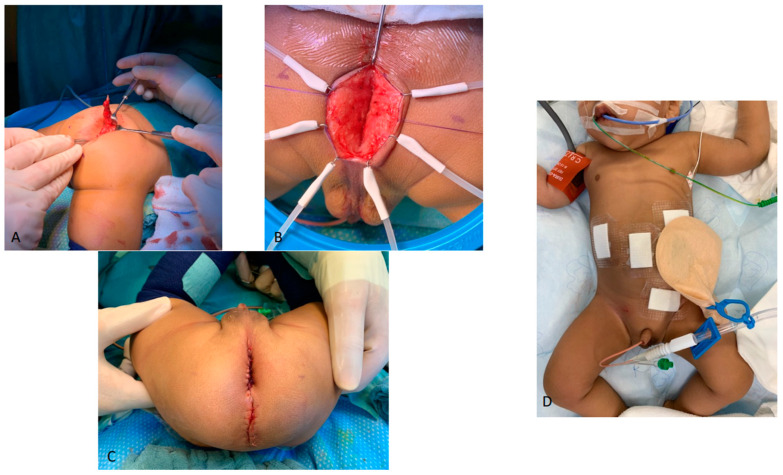
Excision of the meningocele (**A**), first step of the subsequent pull-through (**B**), closing status bottom (**C**), and abdomen (**D**).

At the age of 7 months and a weight of 5.6 kg, we, therefore, planned during the same general anesthesia, the excision of the meningocele first, the laparoscopic ligation of the rectourethral fistula secondly, and then the laparoscopic assisted pull-through with anoplasty (Figure 9). We used a total body preparation so that the patient could be turned alternately in the prone and supine position during the operation. For the meningocele excision performed first, the operation was started primarily in a ventral positioning. We maintained this positioning for the first step of the subsequent pull-through surgery, in which now primarily a vertical skin incision of 3 cm length was performed in the area of the neoanus (spotted using Pena-stimulator), leaving a skin bridge of 1 cm to the wound of the meningocele excision. After the dissection of the muscle complex, we stopped so that the pneumoperitoneum could be preserved during laparoscopy. After repositioning the patient in the supine position, the laparoscopy was performed with a supraumbilically positioned camera and one access in each of the right and left flanks and the left lower abdomen. We confirmed the diagnosis of a cystic formation on the left side of the bladder compatible with a cystic seminal vesicle. The peritoneum was opened all around the distal sigmoid, and the fistula was carefully dissected free. The posterior side of the sigmoid was then detached while sparing and preserving the blood supply to allow a tension-free pull-through. The fistula was then ligated with a Vicryl 5-0 suture. Using the light guidance of the laparoscopic camera, the connecting incision into the abdominal cavity and the subsequent anoplasty could then be performed from the perineal approach. The muscle complex was closed ventrally and dorsally. With a slight tension, the recto-cutaneous anastomosis appeared nicely retracted. It could be easily probed with a Hegar bougie 10. By means of renewed laparoscopy, the sigmoid appeared torsion-free.

The postoperative course was uneventful, and the boy was discharged on day 5. The anoplasty required a regular dilatation from Hegar bougie 7 to 10 until colostomy closure was performed 3 months later. At the age of 18 months, the left orchidopexy, as well as a cystoscopic deflux injection on the right side with circumcision, was performed during the same general anesthesia. In the further care up to the third year of life, the boy showed, under continued physiotherapy, good thriving with good neurological development, problem-free gastrointestinal passage without the occurrence of rectal prolapse, without the occurrence of febrile urinary tract infections with regular clinical-sonographic development of the compensatory growing single kidney and regressive cystic distension of the seminal vesicles.

## 3. Discussion

### 3.1. Discussion Case 1

One-stage total laparoscopic treatment of common bile duct stones in children according to a standardized protocol has been shown to be safe and efficient in centers having the necessary expertise and instrumentation [1]. The intraoperative cholangiogram (IOC) during laparoscopic cholecystectomy (LC) has been advocated to detect intraductal calculi and biliary anomalies undetected by preoperative imaging requiring further treatment in 10% of patients [2]. However, during laparoscopic common bile duct exploration (LCBDE), irrigation with normal saline is sufficient to flush out any stones in most cases, without the need for an additional catheter or endoscopy of the common bile duct [3]. Therefore the laparoscopic common bile duct exploration is a safe alternative to ERCP in the treatment of pediatric choledocholithiasis and is recommended to be a laparoscopic-first approach in the management of choledocholithiasis with IOC to avoid unnecessary procedures in these patients [4]. In an effort to minimize sedation and radiation exposure from fluoroscopy and to provide definitive treatment for choledocholithiasis during a single anesthetic event, some authors have employed dilating balloons via a trans-cystic approach to stretch the sphincter of Oddi with subsequent ductal flushing as a technique of balloon sphincteroplasty [5]. The choice between the trans-cystic duct approach or the choledochotomy approach should be tailored on the basis of information provided by routine intraoperative cholangiography (IOC) and the patient’s biliary tract anatomy. If these criteria are met, the incidence of residual stones is 5%, the complete ductal clearance rate is 95%, and the recurrent stone rate after long-term follow-up is 2% [1]. Furthermore, simultaneous LC and LCBDE in children has not only shown good results and lower morbidity than a two-procedure approach (LC and ERC) [6] but also the advantage of shorter hospital stays and lower cost [6,7].

To further refine this elegant and valuable procedure, we present an illustrated case in which we used the AeroStat rigid tip laparoscopic cholangiography catheter with a percutaneous introducer for an easy and safe intraoperative cholangiography associated with common bile duct stones flushing.

### 3.2. Discussion Case 2

Even though magnets have recently been used successfully as therapeutic agents in pediatric surgery for neonates with high-risk complex esophageal atresia [8,9], they cause great concern in the infant after swallowing because, in combination with a second magnet or a metallic object, they can lead to intestinal perforation with fistula formation and a correspondingly severe course of disease.

If the emergency presentation is prompt and the subsequent radiological workup with a (plain) PA X-ray of the thorax and abdomen and, if swallowing difficulties occur, also a lateral X-ray of the neck is performed directly, the magnets can, in some cases, still be recovered from the pharynx in an ICU setting by using the magnetic attraction of the Magill forceps [10], and in some other cases, they can be retrieved endoscopically from the esophagus or stomach using grasping forceps [11].

However, in any case, swallowed magnets should lead to prompt medical action with consistent controls according to published guidelines [12]. In this approach, a single intestinal and asymptomatic magnet can be examined on an outpatient basis at 24-h intervals by radiologic imaging to determine its motility. However, as soon as multiple asymptomatic magnets are present intestinally, inpatient monitoring with progress radiography should be performed 4–6 h after ingestion. This is in anticipation of the fact that a normal physiologic small bowel passage of 6 h should be assumed, and, accordingly, the magnets should move into the colon within this time. If there is no movement under observation or if the child is symptomatic from the beginning or becomes symptomatic, surgical intervention should be indicated. In a symptomatic child, it does not matter clinically whether one or several magnets are present because, on the one hand, several magnets can simulate the presence of only one magnet in the radiological presentation, and on the other hand, the distance between the magnets may not be apparent if tissue is trapped between them.

If surgery is then indicated, the parents should be asked to provide another of the swallowed magnets so that magnetic strength can be tested preoperatively [13] to avoid an operative trying to search for the magnets in the intestine. In some cases, it might then be noticed that for very strong magnets, metallic instruments are already sufficient to locate the magnets behind the intestinal walls [14]. For smaller magnets, a magnetic laparoscopic instrument is needed, such as the one used in the laparoscopic search for lost needles [15]. Different instruments are mentioned in the publications, ranging from very simple instruments used in laboratory supplies [16] to instruments specifically designed for laparoscopy [17].

In our clinic, we have a simple 6.5 mm diameter and approximately 35 cm long solid magnetic rod, which is a single instrument assigned to the neurosurgical team but is also shared by orthopedic colleagues and our pediatric surgical team. It was manufactured by the Storz company (Tokyo, Japan), but it is no longer commercially available, unfortunately. According to our online research, a corresponding instrument for pediatric surgery that would also fit through a 5 mm trocar does not exist on the market. However, this aspect plays a minor role since the magnets themselves usually have to be removed through a 10 mm trocar due to their diameter, and thus, the magnetic rod can also be designed to fit through a 10 mm trocar. In our example, the seven magnetic beads had a diameter of 5.5 mm. To ensure that the 10 mm trocar with the inserted magnetic rod of approx. 6.5 mm diameter still provides an airtight seal, we slightly slit a 5 mm sealing cap so that the magnetic rod just slides through.

### 3.3. Discussion Case 3

Recently, three-dimensional imaging and printing have been more and more included in everyday clinical practice by a wide range of specialties, including neurosurgery, cardiac surgery, head and neck, plastic or orthopedic surgery, and, more scarcely, pediatric surgery. The goals and uses are multiple, ranging from enhanced anatomical comprehension, teaching, surgical planning, and surgical guidance to implant fabrication and patient communication. [18,19,20,21,22,23,24,25,26]. In our case, we also embedded in the 3D model the pelvic nerve tractography, of interest for pelvic function prognosis in a case of a syndromic anorectal malformation, as described by Muller et al. and Virzi et al. [27,28]. The pelvic nerves were visible and traceable, and we, therefore, concluded that despite the sacral agenesia, the high anorectal malformation, the hypotrophic levator ani, and a very flat bottom, the patient might have a satisfying prognosis regarding urinary and fecal continence. The printed version of the 3D model, including the skin, undoubtedly helped us to visualize the skin incisions required for both the meningocele excision and the laparoscopic rectal pull-through. The most probable context for this case is Currarino syndrome, yet to be genetically proved. Combining a neurosurgical procedure and a pelvic one, in this case, is indeed feasible and advocated by expert centers [29]. The infection risk was also minimized by the presence of the colostomy. However, we decided to take that risk to spare one general anesthesia, with the full understanding of the family, which had been well informed about the surgical plan, possibilities, and risks, thanks to the 3D model. The influence of 3D models on communication quality is currently being explored in our department, using questionnaires before and after the 3D model presentation in patients presenting various types of tumors and malformations.

## 4. Conclusions

In these three cases, we described the possibilities and results of interdisciplinary exchange and transfer of tools, knowledge, and methods between pediatric surgeons and colleagues in other disciplines.

In case 1, we described the modified use, including the adaption to higher safety rules of pediatric surgeries, of an established tool of visceral surgery.

In case 2, we described the transfer of a tool, usually used in open surgery by our colleagues in neurosurgery and orthopedics to find lost metallic particles to a laparoscopic enterotomy for ingested magnets. In case 3, we described how we use 3D printed 3D MRI models, usually used in other application fields, to improve surgical planning and guidance and patient education.

Improving and altering special operations and procedures by transferring knowledge and tools in interdisciplinary setups is wildly evaluated in many fields, but how can we motivate surgeons to spend time and take the extra effort to realize this approach? Are new ideas and methods achieved in formal setups or meetings, or is it more a random walk? All these questions arose during the realization of the described three use cases but still are not answered, and further research could lead to new feasible methods. However, the following idea might be a first step: visiting other teams during operations to see their methods with the exchange of methods in mind. In the case of new observations and ideas, possible alterations of one’s own operations and procedures can be discussed.

## Data Availability

Not applicable.

## Supplementary Materials

The following supporting information can be downloaded at: https://www.mdpi.com/article/10.3390/children9091270/s1, Video S1: cholangiography. Video S2: magnetic retriever.
